# Budesonide/Fomoterol in combination with Montelukast in the treatment of Bronchial Asthma

**DOI:** 10.12669/pjms.36.7.2018

**Published:** 2020

**Authors:** Xiaoxia Dai, Tao Feng, Xuejuan Zhang, Kaishu Li

**Affiliations:** 1 Xiaoxia Dai, Department of Respiratory Medicine, Binzhou People’s Hospital, Shandong, 256600, China; 2 Tao Feng, Department of Ultrasound Medicine, Binzhou People’s Hospital, Shandong, 256600, China; 3 Xuejuan Zhang, Department of Respiratory Medicine, Binzhou People’s Hospital, Shandong, 256600, China; 4 Kaishu Li, Department of Respiratory Medicine, Binzhou People’s Hospital, Shandong, 256600, China

**Keywords:** Bronchial asthma, Budesonide/formoterol, Montelukast, Pulmonary function

## Abstract

**Objective:**

To analyze the clinical effect of budesonide/fomoterol combined with montelukast in the treatment of chronic persistent asthma.

**Methods:**

Ninety-four patients with asthma who came to our hospital for treatment from April 2017 to April 2019 were randomly divided into control group and observation group, with 47 patients in each group. The control group was treated with budesonide/formoterol, and the observation group was treated with montelukast on the basis of the control group. The treatment effect of the two groups was observed and compared.

**Results:**

The total efficacy rate of the observation group was significantly higher than that of the control group (P<0.05); the daytime symptom score and nighttime symptom score of the observation group were significantly higher than those of the control group (P<0.05). The pulmonary function indexes of the two groups after treatment were significantly higher than that before treatment, and the improvement of the observation group was more significant (P<0.05); the FeNO and EO levels of the observation group after treatment were superior to those of the control group, and the difference was statistically significant (P<0.05).

**Conclusion:**

Budesonide/formoterol powder inhalation combined with montelukast can effectively improve the lung function, reduce the level of inflammatory factors, and accelerate the regression of symptoms in the treatment of chronic persistent asthma. It is worth clinical application.

## INTRODUCTION

In recent years, the incidence of bronchial asthma has been increasing. Researches shows that the incidence of bronchial asthma is not caused by a single factor^1^-^3^, and its incidence involves many aspects such as the effects of inflammation and cytokines such as interleukin and tumor factors. The clinical manifestations of bronchial asthma are cough, asthma and dyspnea, which have a serious impact on the quality of life of patients.^4^,^5^ Clinically, bronchial asthma is divided into acute attack period, chronic persistent period and clinical remission period. Previous clinical studies focused on acute attack period. With the improvement of medical environment and medical care, medical staffs have gradually realized the importance of controlling disease condition progress, reducing times of acute attacks and improving the quality of life of patients.^6^,^7^ At present, the treatment of bronchial asthma is mainly based on appropriate drug therapy and compliance. Glucocorticoids are often used to treat bronchial asthma, but the ideal control effect of this kind of drug for some patients is not high, which is not conducive to the prognosis.^8^ Therefore, it is very important for these patients to select the appropriate treatment plan, positively control the progress, and improve the prognosis. In recent years, montelukast has been widely used in clinical practice, but it has not been used in patients with chronic persistent asthma.^9^,^10^ Based on this, the effect of montelukast combined with budesonide/formoterol in the treatment of patients with chronic persistent asthma was evaluated in this study.

## METHODS

Ninety-four patients with chronic persistent asthma after the ethical approval on Oct. 24, 2019 came to our hospital for treatment between April 2017 and April 2019 were selected as the study subjects. They were divided into a control group and an observation group using the method of random number table, 47 each group. In the observation group, there were 26 males and 21 females, aged 25-74 years, with an average age of (51.63±2.62) years; as to the pulmonary function grade, there were 14 mild cases, 18 moderate cases and 15 severe cases; the disease duration was 2~14 years, with an average of (8.41±1.37) years. In the control group, there were 25 males and 22 females, aged 24~76 years, with an average age of (52.31±2.53) years; as to the pulmonary function grade, there were 12 mild cases, 22 moderate cases and 13 severe cases; the disease duration was 2~15 years, with an average disease course of (8.43±1.35) years. There was no significant difference in the clinical data between the two groups (P>0.05); therefore, the results of the two groups can be compared.

### Inclusion criteria were as follows:

1. Patients agreed to be the analysis subjects of the research project, and they or their immediate family members have signed the informed consent.
2. They met the diagnostic criteria of chronic persistent asthma.^11^
3. They had no other respiratory diseases.
4. They were positive in bronchial provocation test or relaxation test.
5. They had complete clinical data, and they could cooperate to complete the research and relevant tests.

### Exclusion criteria included:

1. Being treated by chemotherapy, radiotherapy or immunosuppressive drugs.
2. Having allergic constitution or being allergic to various drugs.
3. Having severe acid-base imbalance and ion disorder.
4. Having symptoms such as fever and other system infections such as pelvic inflammation, peritonetis or urinary tract infection
5. Having severe anemia, cachexia or endocrine disorder.
6. Unable to complete the treatment course or were unwilling to participate in the study.
7. Having cirrhosis or renal insufficiency.

### Therapeutic method

The control group was treated with budesonide/formoterol powder inhalation at a dosage of 160 μg/4.5μg (As-tra Zeneca AB, Sweden, specification: 160 μg/4.5 μg each inhalation), twice a day, for two weeks.

In the observation group, montelukast chewable tablets (Sichuan Otsuka Pharmaceutical Co., Ltd.; H20064828; specification: 5 mg×5 tablets×2 plates) were given orally at a single dose of two tablets, once a day at night, for two weeks.

During the treatment, both groups cooperated with the following nursing protocol. The first nursing care was nursing before atomization inhalation: the ward was kept quiet, clean and at proper temperature; the patients were explained with the role and necessity of atomization inhalation, so as to eliminate the psychological stress and anxiety and maintain a positive and optimistic attitude to cooperate with the treatment; the patients were helped to take a comfortable position to reduce the fatigue of the patients. The second nursing care was nursing during atomization inhalation: before atomization inhalation, the drug was shaken evenly; during atomization inhalation, the patient’s face color and respiratory changes were closely observed, and the patient’s heart rate and blood pressure were observed to see if there was any abnormality. If there was any abnormality, the cause was found out in time. For example, people with too much phlegm were given back clapping treatment. The third nursing care was post treatment of atomization inhalation: after treatment, face washing and mouthwash were given to prevent the continuous and repeated stimulation of the liquid on the face and mouth; the back was pat in time after inhalation to help the patient expel sputum.

### Observation index

The first index was clinical efficacy. Effect was evaluated as significant if the clinical symptoms obviously improved or disappeared, and the lung function related indicators were improved; treatment was evaluated as effective if the clinical symptoms and lung function related indicators of patients changed or improved and as ineffective if the clinical symptoms and lung function related indicators did not change much or intensified. The second index was control condition of disease-related clinical symptoms, which mainly included daytime symptoms and nighttime symptoms scores. Daytime symptoms were evaluated by a six-grade scoring system, zero point for no symptom, one point for transient symptom in the daytime, two points for two or more transient symptoms, three points for symptoms which did not affect daily life in most of the time, four points for symptoms which affected daily life in most of the time, and five points for severe symptoms which made people unable to work and live normally. Night time symptoms were evaluated by a five-grade scoring system, zero point for no symptoms in nighttime, one point for one time of awake at night, two points for two times or more times of awake at night, three points for multiple times of awake at night which made people unable to sleep at most of time, and four points for severe symptoms which made people unable to sleep. The third index was pulmonary function, and the main indexes include forced vital capacity (FVC), forced expiratory volume in one second (FEV1) and FEV1/FVC, which were measured by COSMOD pulmonary function detector. The fourth index was disease-related inflammatory factors, mainly including FeNO and EO. FeNo was detected by Nano Coulomb breath analyzer (Wuxi Shangwo Medical Electronics Co., Ltd., China) and EO by Sysmex analyzer (XS-800i).

### Statistical analysis

Statistical software SPSS21.0 was used to analyze the data. The measurement data were represented by Mean±SD and tested by t test. The counting data was represented by percentage (%) and tested by X^2^ test. The value of P smaller than 0.05 meant difference was statistically significant.

## RESULTS

The total effective rate of the observation group was significantly higher than that of the control group (P<0.05, Table-I).There was no statistically significant difference between the two groups in the scores of daytime and nighttime symptoms before treatment (P>0.05). After treatment, the scores of daytime and nighttime symptoms in the observation group were better than those in the control group, and the difference was statistically significant (P<0.05, Table-II).

**Table-I:** Clinical efficacy between the two groups (%).

Group	Observation group	Control group	X^2^	P
Significantly effective	34(72.34)	23(48.94)	/	/
Effective	9(19.15)	13(27.66)	/	/
Ineffective	4(8.51)	11(23.40)	/	/
Total effective rate	43(91.49)	36(76.60)	9.176	< 0.05

**Table-II:** Improvement of clinical symptoms between the two groups.

	Group	Observation group	Control group
Daytime symptom score	Before treatment	4.24±0.25	4.23±0.22
	After treatment	1.19±0.07^ab^	2.17±0.18^a^
Night symptom score	Before treatment	2.58±0.26	2.59±0.24
	After treatment	38.12±4.53^ab^	46.27±4.18^a^

a *Note:* P<0.05 compared with before treatment;

b P<0.05 compared with 12 hours after treatment.

There was no significant difference in the indexes of lung function between the two groups before treatment (P>0.05). After treatment, FVC, FEV1 and FEV1/FVC in the observation group were better than those in the control group, and the difference was statistically significant (P<0.05, Table-III). After treatment, the levels of FeNO and EO in the observation group were better than those in the control group, and the difference was statistically significant (P<0.05, Table-IV).

**Table-III:** Changes of pulmonary function indexes between the two groups.

	Group	Observation group	Control group	
FVC(L)	Before treatment	2.46±0.07	2.44±0.13
	After treatment	1.17±0.16	2.66±0.66^a^
FEV1(L)	Before treatment	1.87±0.84^ab^	1.13±0.18
	After treatment	38.12±4.53^ab^	1.40±0.23^a^
FEV1/FVC (%)	Before treatment	71.83±6.34	70.03±6.16
	After treatment	89.59±10.23^ab^	80.11±10.73^a^

a *Note:* P<0.05 compared with before treatment;

b P<0.05 compared with 12 hours after treatment.

**Table-IV:** Improvement of disease-related inflammatory factors in two groups.

	Group	Observation group	Control group
FeNO(ppd)	Before treatment	39.17±3.16	39.13±3.18
	After treatment	15.73±2.12^ab^	20.43±2.23^a^
EO(%)	Before treatment	9.57±2.83	9.54±2.79
	After treatment	5.61±2.04^ab^	7.27±2.12^a^

*Note:* ^a^P<0.05 compared with before treatment; ^b^P<0.05 compared with 12 hours after treatment.

## DISCUSSION

Chronic persistent bronchial asthma is common in clinic, which often manifests symptoms such as chronic cough and shortness of breath. With the development of chronic persistent bronchial asthma, the pulmonary function of the patients is obviously decreased, and respiratory failure often occurs.^12^ Related studies show that budesonide/formoterol inhalation could improve the clinical symptoms of patients with chronic persistent bronchial asthma, but it is not ideal for improving lung function and disease-related inflammatory factors due to many reasons, mainly manifested in that budesonide/formoterol inhalation had the function of immunosuppression. Especially for patients with low immune function, this treatment scheme cannot significantly improve the prognosis, which will affect the clinical treatment effect.^13^

In recent years, with the improvement of clinical technology, the treatment of chronic persistent asthma has also improved. For example, the application of montelukast which. is a highly selective leukotriene receptor antagonist, which can block the process of leukotriene binding receptor, inhibit asthma attack, suppress the synthesis and release of a variety of inflammatory mediators, promote the expression of a variety of anti-inflammatory factors, regulate the level of T-lymphocytes and natural killer cells (NK) and the immune imbalance. It also promote the improvement of lung function.^14^,^15^ A relevant study show that the pharmacodynamics of montelukast tends to be stable within one day without tolerance, and it can effectively relieve cough, asthma and other uncomfortable symptoms.^16^ Rajanandh et al. found that the therapeutic effect of therapy of budesonide/formoterol combined with montelukast was significant in the treatment of asthma, superior to other combined therapies, and the patients had good tolerance.^17^ The results of this study also suggested that the total effective rate and the improvement of pulmonary function of the observation group treated with budesonide/formoterol and montelukast were better than those of the control group, which were in line with the above results. The results of this study also showed that the clinical symptom scores of daytime and nighttime of the observation group were better than those of the control group, similar to the finding of Ding.^18^

In addition, the research of Li et al. also proposed that patients with bronchial asthma who were treated with montelukast combined with budesonide/formoterol had good clinical prognosis, and it effectively improved the biochemical indicators of patients.^19^ However, there are few reports about the effect of combined therapy on inflammatory factors. The results of this study showed that budesonide/formoterol inhalation combined with montelukast could significantly reduce the levels of FeNO and EO in patients with chronic persistent bronchial asthma. EO is one of the main inflammatory factors inducing asthma, almost involved in the whole course of asthma inflammation reaction, and it can also induce airway hyperresponsiveness. The high level of serum in patients with asthma is mainly related to the immune response of allergens.^20^ EO can cause bronchospasm by releasing histamine, and moreover it can also damage the mucosal barrier and cells to a certain extent, leading to airway mucosal edema and a series of symptoms and signs. FeNO is produced by airway cells, and its concentration and content are related to the level of inflammatory factors; it is an important marker to evaluate the control of asthma as it can reflect the level of airway EO inflammation.^21^ It shows that the combination therapy has a good anti-inflammatory effect and can effectively inhibit the EO level, control the development of the disease, reduce the stimulation of inflammatory factors, and relieve the damage to the airway mucosa, which can help to improve the airway reconstruction and airway hyperresponsiveness and reduce the times of acute attacks.

## CONCLUSION

The combination of budesonide/formoterol inhalation and montelukast has a good clinical effect in the treatment of chronic persistent bronchial asthma which can significantly improve the lung function, inhibit the level of related inflammatory factors, relieve the clinical symptoms, enhance the quality of life, and promote the prognosis. It can be promoted in clinics.

### Authors’ Contribution:

**XXD & KSL:** Study design, data collection, and analysis and is responsible for integrity of study.

**XXD, TM & XJZ:** Manuscript preparation, drafting and revising.

**KSL:** Review and final approval of manuscript
